# Touched by your words: How touch-related vocabulary prompts charitable behavior by reducing the negative effect of disgust

**DOI:** 10.3389/fpsyg.2023.1104356

**Published:** 2023-03-07

**Authors:** Olivia Petit

**Affiliations:** Marketing and New Consumption Center of Excellence, Kedge Business School, Marseille, France

**Keywords:** Midas Touch Effect, disgust, trust, empathy, charity, social marketing

## Abstract

Getting help is often difficult for people who trigger disgust (e.g., homeless, sick, or disabled people) as well as the charities representing them because of low trust in these groups. Prior research has demonstrated that physical contact can help increase generosity. However, it is difficult to trigger this phenomenon—called Midas Touch Effect—when people feel disgust and are uncomfortable with interpersonal touch. This research examines touch-related vocabulary (e.g., “I would be touched,” “anyone who I can contact”) as an alternative, non-physical way for prompting the Midas Touch Effect. This research examines if such a vocabulary may reduce the negative effects of disgust on trust, and thus increase the willingness to donate. Across two studies, it is shown that while disgust has a negative effect on trust and willingness to donate to a homeless person when no touch-related vocabulary is used, no such negative effect is observed when the message includes touch-related vocabulary.

## 1. Introduction

Low-status groups such as the homeless (Rozin et al., 2008) can trigger disgust because they are often associated with a lack of hygiene or mental illness (Clifford and Piston, 2017). Such disgust represents an important issue for charities to the homeless, because it decreases the trust that is important for giving (Aarøe et al., 2016; Alhidari et al., 2018). Furthermore, giving is important to fill the missing funds for vital homelessness services (over £1 billion short in the United Kingdom, Thunder and Rose, 2019).

Disgust also incites people to keep their distance with people judged disgusting (Harris and Fiske, 2007), and who have no opportunity to elicit touch with potential givers. Touch conveys trust from one person to another, makes people more likely to accept a request, and even sacrifice money (Crusco and Wetzel, 1984; Gallace and Spence, 2010). It also increases social proximity (Lähdesmäki and Suutari, 2012), and has positive effects on willingness to donate (Ghorbani et al., 2013). This positive effect of touch on prosocial behavior is referred to as the Midas Touch Effect (Crusco and Wetzel, 1984) –inspired by King Midas from Greek mythology, who turned everything he touched into gold.

Due to the disgust elicited by certain low-status groups, individuals often want to avoid physical contact (Sussman and Rosenfeld, 1982), thus limiting the occurrence of the Midas Touch Effect. Nevertheless, this research demonstrates that the use of touch-related vocabulary can positively affect trust and the willingness to donate by verbally triggering the Midas Touch Effect.

## 2. Theoretical background

### 2.1. Trust and disgust

Social trust can be defined as the belief that others will not voluntary do us harm, if they can avoid it (Delhey and Newton, 2005). It is different from trust in individuals, which concerns familiar people (Uslaner, 2002). Social trust is important because it influences collective action (Uslaner, 2002), and has been shown to be particularly relevant for giving to charity (Alhidari et al., 2018). However, several studies have shown that social trust can be affected by disgust (Aarøe et al., 2016; Kugler et al., 2020), which could be harmful for the homeless.

Over the evolution, our behavioral immune system has been developed to scan for potential pathogen threats (Aarøe et al., 2017). When a potential threat is identified, disgust is activated. Disgust is a basic emotion characterized by facial expressions and physiological manifestations that can be seen as signals facilitating the activation of avoidance behaviors (Rozin et al., 2008). As the system is not perfectly calibrated, it tends to treat any cue of disease as a potential threat (Aarøe et al., 2017). Homeless people often lack access to healthcare and suffer from high rates of illness (Gelberg et al., 1990). They therefore constitute a perceived threat of pathogens and are likely to trigger disgust (Clifford and Piston, 2017).

Disgust triggered by other people is not only used as a barrier against physical threats, it also helps to protect the social order (Tybur et al., 2013). People generally perceive strong symbolic associations between physical and moral purity (Liljenquist et al., 2010). In this regard, disgust is often associated with harsh moral judgment (Schnall et al., 2008). Conversely, trust is conceptualized as a moral behavior (Wicks et al., 1999). For this reason, disgust has often been a brake on trust (Aarøe et al., 2016; Kugler et al., 2020), which may explain the lack of trust often observed toward homeless people (Cikara et al., 2010).

### 2.2. Touch word as a moderator

Interpersonal touch refers to non-verbal behavior that is often considered to be an expression of attachment, emphasizing a social proximity between individuals (Knapp, 1978). This tactile contact increases trust (Orth et al., 2013), and acts of generosity (Crusco and Wetzel, 1984). This positive effect of touch on prosocial behavior is called the Midas Touch Effect (Crusco and Wetzel, 1984). It is linked to biological underpinnings, according to which contact comfort is a motivating agent for affectional responses (Gallace and Spence, 2010). From this background, interpersonal touch is usually seen as producing warmth and comfort (Webb and Peck, 2015).

In line with the aforementioned positive effect of touch on trust (Orth et al., 2013), Morhenn et al. (2008) found that interpersonal touch, when followed by an act of trust, leads people to sacrifice money for others. Thus, trust might play an important role in the effect of interpersonal touch on donations to homeless people. However, the problem is that potential donors tend to limit physical contact with homeless people because they trigger disgust (Clifford and Piston, 2017).

What it is suggested that because actual physical contact might elicit disgust and will therefore be difficult to establish with homeless people, the use of touch-related words could substitute interpersonal touch and have the same positive effects on generosity. According to neuroscience research, sensory imagery can activate areas of the brain corresponding to the sensory experience to produce similar sensations (Simmons et al., 2005; Barsalou, 2008), positively affecting consumer behavior (Petit et al., 2018, 2022). Sensory imagery can be based on images and also on words (Simmons et al., 2005; González et al., 2006). Peck and Wiggins (2006) demonstrated that using touch-related words (e.g., get in touch) in a charity pamphlet can produce strong affective responses to the message, which in turn increased the willingness to donate to the charity. Therefore, the use of touch-related words may reduce the negative effect of disgust toward homeless people on trust, leading to an increase in generosity. Thus, it is postulate that:

*Hypothesis 1*: Touch-related vocabulary reduces the negative effect of disgust on trust.

*Hypothesis 2*: Touch-related vocabulary reduces the negative indirect effect of disgust on the willingness to donate through trust.

The following studies involving human participants were reviewed and approved by Kedge Business School ethics committee. Written informed consent to participate in this study was provided by the participants.

## 3. Study 1

### 3.1. Stimuli and procedure

Study 1 aimed to demonstrate that disgust negatively affects trust when there is no touch related vocabulary in the message, while no such negative effect of disgust was to be observed when a touch-related vocabulary was used. A total of 420 participants from United Kingdom (69.4% female, *M_age_* = 33.86, *SD* = 10.6) were recruited through Prolific, and took part in a 2 (vocabulary: touch vs. control) × 2 (disgust: high vs. low) between-subjects online experiment. 14 participants were removed because they did not give a correct answer to the attention-check (select “strongly disagree”).

Participants were exposed to an image of a fundraising advertisement for homeless people. The four versions of the advertisement presented the same image of a homeless person and a different text for each condition. The presence versus the absence of a touch-related vocabulary was manipulated in the sentences used by the homeless person asking for money (see Supplementary material). Disgust was manipulated in the description of the homeless person.

Participants were randomly exposed to the stimulus and then completed a three-item measure of trust toward the homeless person, adapted from Nicholson et al. (2001) (α = 0.85). Next, participants rated the disgust elicited by the homeless person (i.e., “To what extent would you feel disgusted by this man,” on a seven-point Likert scale, adapted from Tybur et al., 2009). Arousal (calm, relaxed, sleepy—excited, activated, vigilant) and valence (unpleasant, unhappy, angry—pleasant, happy, delighted) were controlled, by asking participants to complete the Self-Assessment Manikin on nine-point Likert scales (Mehrabian and Russell, 1974).

### 3.2. Results

It was first checked that the participants rated feeling more disgust in the high disgust conditions than in the low disgust conditions [*M_low_* = 1.81, *SD =* 0.98, *M_high_* = 2.86, *SD =* 1.4, *t*(404) = −8.77, *p* < 0.001, −1.05, 95% *CI*[−1.28, −0.81]]. It was also ensured that using a touch-related vocabulary did not change the emotions triggered by the message, for arousal [*M_control_* = 4.86, *M_touch_* = 4.88, *t*(404) = −1.51, *p* = 0.88, −0.02, 95% *CI*[−0.28, 0.24]] and valence [*M_control_* = 3.45, *M_touch_* = 3.56, *SD =* 1.29, *t*(404) = −0.71, *SD =* 1.34, *p* = 0.48, −0.11, 95% *CI*[−0.41, 0.19]].

**Figure 1:**
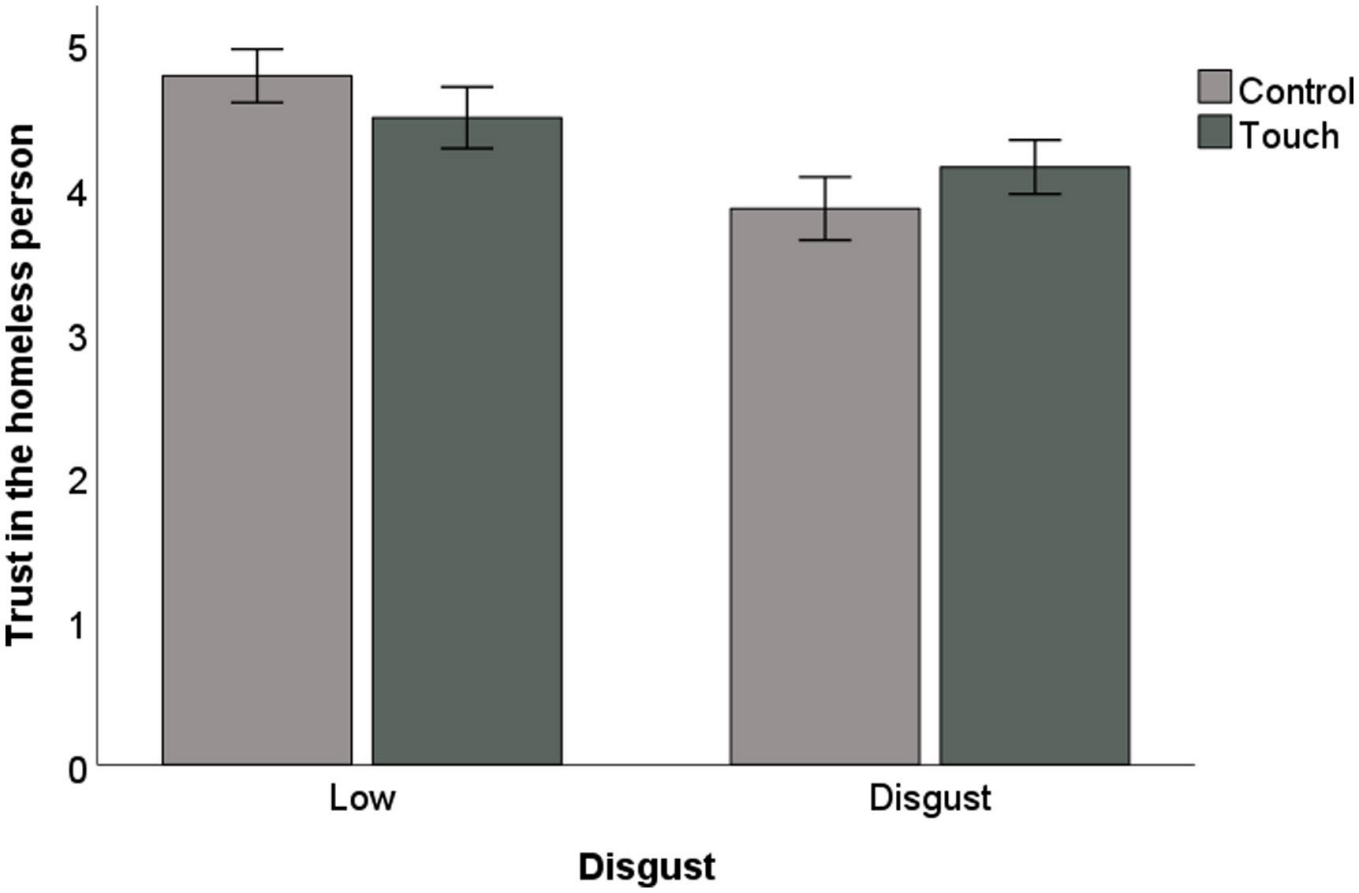
Study 1: Trust as a function of disgust and touch-related vocabulary.

In order to test H1, a two-way ANOVA was conducted with disgust (coded as a dummy variable: 0 = low, 1 = high) and touch-related vocabulary (coded as a dummy variable: 0 = control, 1 = touch) as independent variables and trust as dependent variable. Results revealed a main negative effect of disgust [*F*(3,402) = 38.64, *p* < 0.001], and no main effect of the touch-related vocabulary (*p* = 0.99). Importantly, results yielded a significant interaction between disgust and touch-related vocabulary on trust [*F*(3,402) = 8.48, *p* = 0.01]. *Post hoc* Bonferroni-corrected tests revealed that in the control conditions, trust was higher in the situation of low disgust (*M*_low_ = 4.78, *SD =* 4.76 vs. *M*_high_ = 3.86, *SD =* 1.1; *p* < 0.001). Similar results were found in the touch-related vocabulary conditions (*M*_low_ = 4.49, *SD =* 1.09 vs. *M*_high_ = 4.14, *SD =* 0.94; *p* < 0.02). For one in the situation of low disgust, trust was higher in the control condition (*M*_control_ = 4.78, *SD =* 4.76 vs. *M*_touch_ = 4.49, *SD =* 1.09; *p* = 0.04). For one in the situation of high disgust, trust was higher when a touch-related vocabulary was used (*M*_control_ = 3.86, *SD =* 1.1 vs. *M*_touch_ = 4.14, *SD = *0.94; *p* = 0.05; Figure 1). These results indicated that even though trust remained lower in a condition of high disgust than in a condition of low disgust, regardless of the vocabulary used, such negative effects of disgust were reduced when a touch-related vocabulary was used, providing support to H1.

## 4. Study 2

### 4.1. Stimuli and procedure

The objective of Study 2 was to demonstrate that touch-related vocabulary can increase trust and consequently willingness to donate when disgust is high (vs. low). In this study, participants had to imagine themselves directly interacting with the homeless person rather than being exposed to an advertisement (Edell and Staelin, 1983; Lewis et al., 2013). Based on the results of Study 1, sample size calculations were performed with an alpha of 0.05 and 80% power. A total of 132 participants from United Kingdom (48.5% female, *M_age_* = 29.33, *SD* = 8.57) were recruited through Prolific and took part in a 2 (disgust: high vs. low) × 2 (vocabulary: touch-related vs. control) between-subjects online experiment. Participants were presented with an online scenario depicting a situation in which they encountered a homeless person. The scenarios were similar to Study 1, and manipulated the presence vs. the absence of a touch-related vocabulary in the sentences used by the homeless person. Disgust was manipulated in the description of the homeless person. Participants were asked to imagine having 10 Euros in their wallet before indicating the amount of money they would be willing to donate to the homeless person (adapted from Ein-Gar and Levontin, 2013; *M* = 2.16, *SD* = 2.09). Then, participants completed the three-item measure of trust (α = 0.88). Finally, participants rated the disgust elicited by the homeless person on a seven-point Likert scale (adapted from Tybur et al., 2009).

### 4.2. Results

It was first checked that the participants rated feeling more disgust in the high disgust conditions than in the low disgust conditions [*M_Low_* = 2.21, *SD =* 1.16, *M_High_* = 4.38, *SD =* 1.5, *t*(64) = −6.54, *p* < 0.001, −2.16, 95% *CI*[−2.82, −1.5]]. In order to test H1, a two-way ANOVA was conducted with disgust and touch-related vocabulary as independent variables and trust as dependent variable. Results revealed no main effect of the touch-related vocabulary [*F*(1,128) = 0.248, *p* = 0.62] and disgust [*F*(1,128) = 2.94, *p* = 0.09], but—as predicted—a significant interaction between disgust and touch-related vocabulary [*F*(1,128) = 5.37, *p* = 0.02] on trust. In order to better understand this interaction, a *t*-test was performed in each of the two touch-related vocabulary conditions. In the control condition, results revealed a significant negative effect of disgust on trust (*M*_Low_ = 4.28, *SD =* 1.02, *M*_High_ = 3.47, *SD =* 1.2, *p* = 0.01), whereas this effect was not found in the touch-related vocabulary condition (*M*_High_ = 4.04, *SD =* 1.16, *M*_Low_ = 3.91, *SD =* 1.19, *p* = 0.68). As in Study 1, these results therefore showed that a touch-related vocabulary reduced the negative effect of disgust on trust. Overall, these results supported H1.

**Figure 2:**
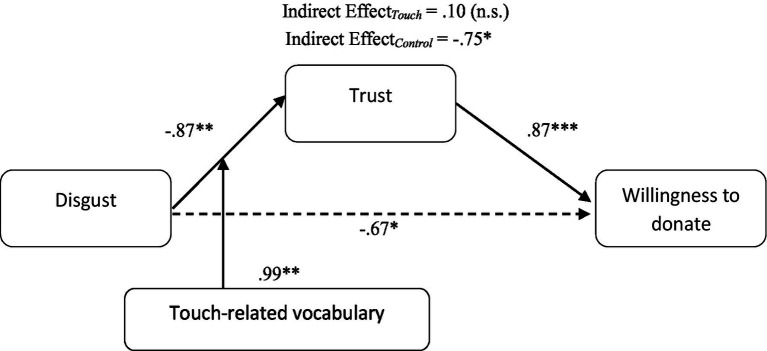
Study 2: The mediating role of trust in the interacting effect of disgust and touch-related vocabulary on willingness-to-donate.

Next, in order to test H2, a moderated-mediation analysis was performed (PROCESS v3.1, Model 7, 5,000 bootstraps). Specifically, disgust was included as the independent variable (coded as a dummy variable: 0 = low, 1 = high), trust as the mediating variable, willingness to donate as the dependent variable, and the touch-related vocabulary conditions as the moderating variable (coded as a: 0 = control, 1 = touch). Results revealed a significant index of moderated-mediation (Index = 0.865, *S.E*. = 0.363, *95% CI* = 0.175; 1.637). As hypothesized, the results revealed a significant negative indirect effect of disgust on willingness to donate through trust when no touch-related vocabulary was employed (Effect = −0.756, *S.E*. = 0.273, 95% *CI* = [−1.328; −0.254]). In this condition, disgust exerted a negative effect on trust (−0.87, *t* = −3.13, *p* = 0.01), which in turn increased the willingness to donate (1.04, *t* = 5.01, *p* < 0.001). In contrast, the touch-related vocabulary condition showed a lack of such negative indirect effect (Effect = 0.100, *S.E*. = 0.252, 95% *CI* = [−0.411; 0.602]; Figure 2). These results supported H2 and showed that beyond trust, willingness to donate was influenced by the interaction of touch-related vocabulary and disgust.

## 5. Discussion

### 5.1. Summary

This research set out to examine the potential of using a touch-related vocabulary to trigger the Midas Touch Effect and reduce the negative effect of disgust on trust and willingness to donate. The results of two studies demonstrated that using such a vocabulary in a fundraising advertisement (Study 1), or directly by the requester (Study 2) could be particularly useful to trigger the Midas Touch Effect in situations of disgust. Study 1 showed that the negative effect of disgust on trust toward a homeless person was not observed when a touch-related vocabulary was employed. Study 2 extended these results by showing that touch-related vocabulary moderated the indirect effect of disgust on the willingness to donate through trust.

### 5.2. Theoretical and practical implications

This study makes several contributions to the literature on prosocial behavior, especially regarding homeless people. First, this study highlights the negative effect of disgust on trust toward homeless people and its impact on willingness to donate. Clifford and Piston (2017) showed that disgust generated by homeless people motivates the desire for physical distance, leading to support for policies that exclude homeless people from public life. Cikara et al. (2010) found that disgust and low warmth associated with homeless people increased their participants’ willingness to sacrifice the homeless for the good of the community. This research suggests that these aversive behaviors toward homeless people can be explained by the negative effect of disgust on trust (Aarøe et al., 2016; Kugler et al., 2020). This research shed new light on the act of donation by showing that disgust can make it more difficult. Disgust generates a need for physical (pathogen treat) and social (moral judgment) distances from the potential donors (Harris and Fiske, 2007; Winterich et al., 2015), which leads them to develop negative perceptions of the requesters (Park et al., 2007; Aarøe et al., 2016), and thus making them less willing to donate.

Second, in search of a way to mitigate the negative effects of disgust, this research builds on the Midas Touch Effect literature to show that touch-related vocabulary can result in prosocial behavior (Gallace and Spence, 2010). The Midas Touch Effect, elicited through interpersonal touch, has been shown to influence trust and monetary sacrifice toward strangers (Morhenn et al., 2008; Rodrigues et al., 2009). Unfortunately, interpersonal touch is not possible from a distance, so it is difficult to produce the Midas Touch Effect in situations of disgust. This is also the case during social interactions on the Internet where people may be asked to donate online. Previous research revealed that the Midas Touch Effect is difficult to reproduce online through digital interfaces because of the lack of physical contact (Gallace and Spence, 2010; Petit et al., 2019). Therefore, this research is the first to demonstrate the possibility of creating a Midas Touch Effect without physical contact through the use of a touch-related vocabulary and showing its effects on prosocial behavior. It opens the way to new research for charity on the use of touch-related words in donation campaigns, and more generally on situations of social interaction without physical contact.

Third, this research identifies the conditions under which touch-related vocabulary can trigger the Midas Touch Effect, as well as the variables on which it acts to produce prosocial behavior. It was found that this vocabulary is only effective in situations that involve a certain extent of perceived disgust. This result can be explained by the fact that the Midas Touch Effect is generally observed in situations where trust is challenging (Morhenn et al., 2008). Indeed, disgust has been shown to have a direct negative effect on social trust and therefore constitutes a particularly relevant context for triggering the Midas Touch Effect (Aarøe et al., 2016; Kugler et al., 2020). These results imply that touch-related vocabulary should be incorporated into messages aimed at triggering donations when the subjects in the communication elicit disgust (e.g., sick, disabled, elderly, and destitute).

### 5.3. Limitations and research perspectives

The current research has some limitations. First, in Study 1, an unexpected negative effect of touch-related vocabulary on trust was found in the low disgust condition. It is possible that the lack of disgust created a greater closeness between the participants and the homeless, and that the touch-related vocabulary led to a fear of contagion. It has been shown that in a situation of proximity, imagined touch can have a “contagion effect” and elicit disgust (Morales and Fitzsimons, 2007). Further studies are needed to understand these relationships. Second, this research focused on the willingness to donate and did not measure actual donations. Therefore, actual giving measures should be considered. Third, this research examined the effects of touch-related vocabulary in a context of disgust that was limited to homeless people. However, different groups of people could potentially elicit disgust, like those that possess features that connote disease (Oaten et al., 2009) or the elderly (Karlsson and Gunnarsson, 2018). Future research could examine if the use of a touch-related vocabulary could elicit more positive reactions and increase donations to these specific targets. Finally, emotions such as sadness, or guilt can also prompt charitable behavior (Ongley et al., 2014; Homer, 2021). One may therefore wonder to what extent touch-related vocabulary might help in situations where requests for money are made by people or organizations associated with other emotions.

## Data availability statement

The raw data supporting the conclusions of this article will be made available by the authors, without undue reservation.

## Ethics statement

The studies involving human participants were reviewed and approved by Kedge Business School ethics committee. Written informed consent to participate in this study was provided by the participants.

## Author contributions

OP contributed to conception and design of the study and manuscript revision, performed the statistical analysis, wrote the first draft of the manuscript, and read and approved the submitted version.

## Conflict of interest

The author declares that the research was conducted in the absence of any commercial or financial relationships that could be construed as a potential conflict of interest.

## Publisher’s note

All claims expressed in this article are solely those of the authors and do not necessarily represent those of their affiliated organizations, or those of the publisher, the editors and the reviewers. Any product that may be evaluated in this article, or claim that may be made by its manufacturer, is not guaranteed or endorsed by the publisher.
