# Supplementary figures and images for: Touched by your words: How touch-related vocabulary prompts charitable behavior by reducing the negative effect of disgust
Source: Front Psychol. 2023 Mar 7;14:1104356. doi: 10.3389/fpsyg.2023.1104356 (PMC10029352; doi:10.3389/fpsyg.2023.1104356)

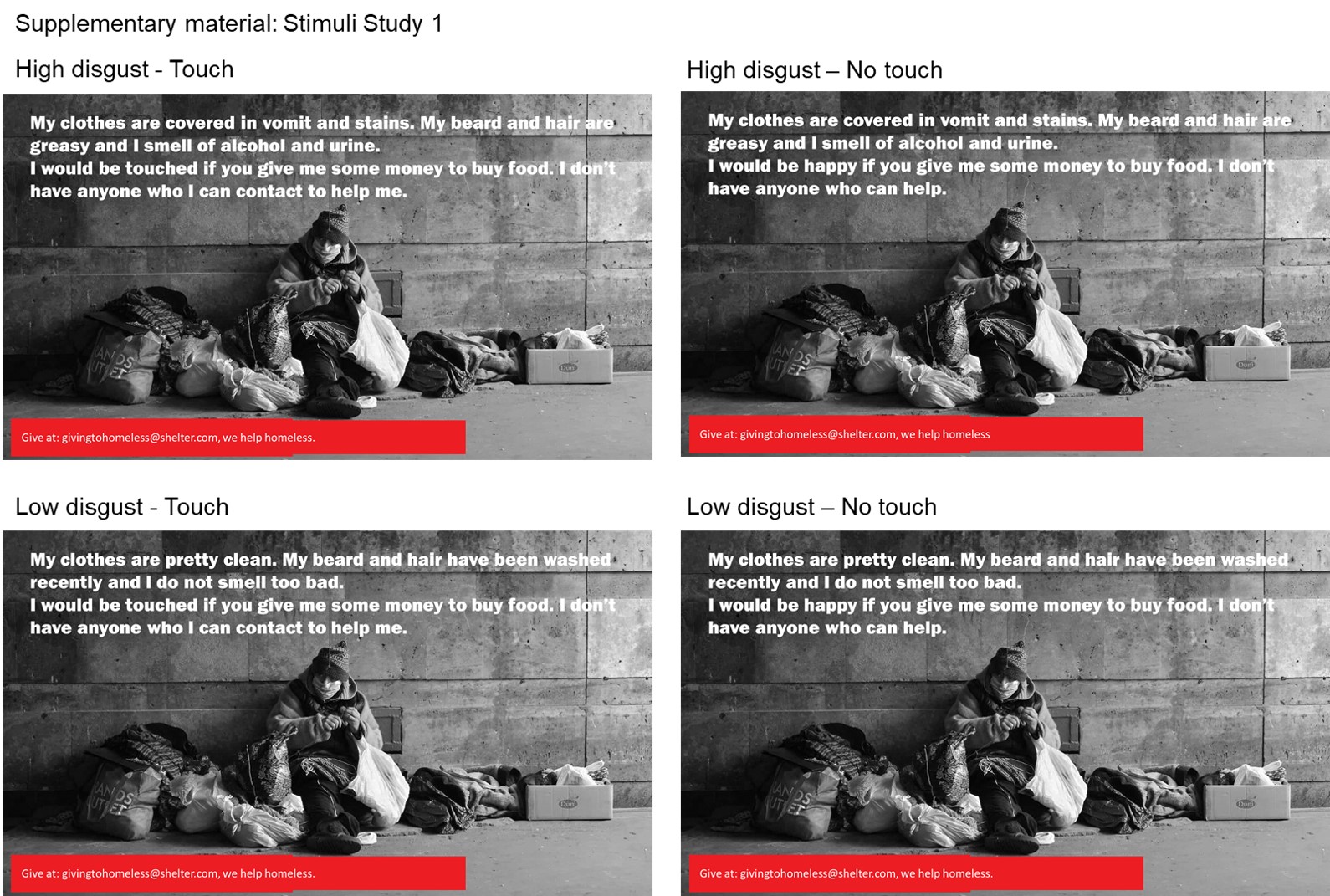

Supplement: Supplementary file 1 [file Image_1.jpg]
